# Editorial: Education and training for laboratory automation

**DOI:** 10.1155/S1463924688000136

**Published:** 1988

**Authors:** Peter B. Stockwell


Journal of Automatic Chemistry, Vol. 10, No. 2 (April–June 1988), p. 65

### Editorial: Education and training for laboratory automation

Laboratory automation has become so commonplace over the last few years that it is widely accepted and fully integrated into our university graduate teaching programmes, but is it really? When we started the *Journal* in 1978, it was clear that few colleges were in fact teaching laboratory automation and that, particularly in the UK, the basic training for our analysts provided little, if any, background in electronics and/or computing. One of our founding editorial advisers, Professor Howard Malmstadt, had been pioneering the field in the USA and his foresight has created a hierarchy of teachers, who instruct on aspects of automation to various groups. In *Analytical Chemistry*, Vol. 60, No. 2 (15 January 1988) p. 87A ff, he was rightly honoured for his contribution. The article includes a family-tree of Malmstadt students – a pretty impressive group doing valuable work. They are however, only focusing on areas of automation and still do not focus any attention on automation proper. Furthermore, no effort at all seems to be given to the most difficult and time-consuming area of sample preparation. Generally, this group has given most attention to the role of electronics and computing in chemistry.

In the UK, a number of university groups were founded with the intention of researching into aspects of instrumentation and automation. Sadly, these have not really flourished – the group at the University of Manchester Institute of Science and Technology (UMIST), for example, suffered by the untimely death of Gordon Kirkbright and the Swansea group, into which my former employer, the Laboratory of the Government Chemist, had invested time and funding is now no more. The attraction of an industrial, as opposed to academic, salary being the cause of this situation. The failure to replace both groups has left a void in the teaching programme. Had automation become so widely used and understood that a teaching programme was now no longer needed?

Obviously chemical analysis has changed and involves automation. Therefore it must of course be included in the undergraduate teaching programme to some degree. Few university professors seem to be able to include it and are only teaching the barest essentials of philosophy of automation.

*Journal of Automatic Chemistry* covers all aspects of automation including economic justifications and training. The subject area is progressing at a fast rate, but the lessons learned in the past and the general background are invaluable before a system's designer should embark on a new project.

Some years ago I was actively involved in a short course on laboratory automation. The course's programme has been described previously in the *Journal*. Whilst the full scope of this course would need to be rebuilt to include latest developments, several of its more fundamental aspects would find use as the general core of the requirement for today's likely students. Maybe people who use automatic systems on a day-to-day basis do not feel the need for such a course – but only if their analytical needs are restricted should this really be the case. New recruits can quickly become useful, given access to such background information as given in these courses. Hopefully, a new group of people will be forthcoming to revitalize such courses, and I can assure any potential organizers that the return on time and investment from a technology point of view is very high.

When we published the titles *Automatic Chemical Analysis* and *Topics in Automatic Chemical Analysis*, in 1974 and 1979, respectively (published by Ellis Horwood, Chichester), we saw these as forerunners of a series of continuing updates. Sadly, this has not been possible and few books have appeared which cover the subject in any broad detail. It is important that such topics are brought up-to-date and communicated to a wider audience.

In future issues of the *Journal*, we hope to be able to provide valuable tutorial aids to the philosophy and practice of laboratory automation in its widest sense. We hope that members of the present editorial board and new recruits to it will be able to provide such material. Whilst accepting that these people are themselves extremely busy, I hope that we can persuade them that this will be a valuable contribution, not only to students about to start a career in analysis, but as a start too, on the job training for scientists joining teams at the forefront of developments in automatic analyses.

I look forward to receiving your comments and suggestions on this and other topics.

**Peter B. Stockwell**

